# High-Frequency Vibration Treatment of Human Bone Marrow Stromal Cells Increases Differentiation toward Bone Tissue

**DOI:** 10.1155/2013/803450

**Published:** 2013-03-25

**Authors:** D. Prè, G. Ceccarelli, L. Visai, L. Benedetti, M. Imbriani, M. G. Cusella De Angelis, G. Magenes

**Affiliations:** ^1^Department of Industrial and Information Sciences, University of Pavia, Via Ferrata 1, 27100 Pavia, Italy; ^2^Center for Tissue Engineering, University of Pavia, Via Ferrata 1, 27100 Pavia, Italy; ^3^Department of Public Health, Experimental Medicine and Forensics, University of Pavia, Via Forlanini, 8, 27100 Pavia, Italy; ^4^Department of Molecular Medicine and UdR INSTM, University of Pavia, Viale Forlanini 6, 27100 Pavia, Italy; ^5^Laboratory of Nanotechnology, Salvatore Maugeri Foundation IRCCS, Via S. Maugeri 4, 27100 Pavia, Italy

## Abstract

In order to verify whether differentiation of adult stem cells toward bone tissue is promoted by high-frequency vibration (HFV), bone marrow stromal cells (BMSCs) were mechanically stimulated with HFV (30 Hz) for 45 minutes a day for 21 or 40 days. Cells were seeded in osteogenic medium, which enhances differentiation towards bone tissue. The effects of the mechanical treatment on differentiation were measured by Alizarin Red test, (q) real-time PCR, and protein content of the extracellular matrix. In addition, we analyzed the proliferation rate and apoptosis of BMSC subjected to mechanical stimulation. A strong increase in all parameters characterizing differentiation was observed. Deposition of calcium was almost double in the treated samples; the expression of genes involved in later differentiation was significantly increased and protein content was higher for all osteogenic proteins. Lastly, proliferation results indicated that stimulated BMSCs have a decreased growth rate in comparison with controls, but both treated and untreated cells do not enter the apoptosis process. These findings could reduce the gap between research and clinical application for bone substitutes derived from patient cells by improving the differentiation protocol for autologous cells and a further implant of the bone graft into the patient.

## 1. Introduction

Despite an increased interest in tissue regeneration, the availability of cells remains a serious limitation for clinical applications in regenerative medicine. Autologous adult stem cells must be expanded and induced along specific cell lines; this process implies long culture periods. Thus, a great challenge consists in finding methods to selectively improve differentiation toward tissue, which would consequently reduce the differentiation period. Several stimulation methods that use different sources of energy have been widely tested to improve the rate of differentiation of primary cell culture to osteoblasts, that is, ultrasound [1, 2] and electromagnetic fields [3–5]. In particular, following a biomimetic approach, mechanical stress has been demonstrated to be a good stimulus. It promotes early cell differentiation into bone [6, 7] and may be achieved by either flow perfusion bioreactors [6, 8] or physical mechanical stress [9, 10]. In fact, cells such as osteoblasts and fibroblasts respond to a variety of stresses, including compression, torsion, and tension. These facilitate matrix turnover and remodelling [11, 12] through mechanical signals which are translated by cell surface receptors via intracellular pathway activation and respond to the duration and intensity of the stimulation with several outcomes, including cell proliferation, differentiation, and apoptosis [13–15]. In particular, stimulation with high-frequency vibration (HFV) has provided positive results on *in vivo* bone tissue affected by bone diseases [16–18] and it has been demonstrated that high-frequency, mechanical vibrations stimulate the *in vitro* differentiation of SAOS-2 cells [19] and human adipose-derived stem cells (hASCs) [20] toward bone tissue. Moreover, potent biochemical mediators such as growth factors also affect cell development and repair processes through similar intracellular activation pathways [21, 22]. 

For more than twenty years, BMSCs (bone marrow stromal cells) have been considered a good source of osteoblast precursor cells [23]. Recent studies have demonstrated the differentiation potential of BMSC. With the appropriate culture medium and conditions, these human stem cells can differentiate into ligament, tendon [24, 25], muscle [26, 27], nerve [28, 29], endothelium [30, 31], or hepatic tissue [32, 33]. Therefore, we decided to treat BMSC with HFV in order to determine whether the promising results obtained with SAOS-2 and hASC could be extended to human bone-marrow-derived stem cells. We tested the effects of HFV at two phases of BMSC differentiation toward osteocytes: after 21 days (as tested for hASC [34]) and at 40 days to verify the terminal differentiation state, which has been demonstrated to be around 35–40 days [34, 35] for BMSC.

Previous studies have demonstrated the osteogenic effects of supplemental chemical factors, such as dexamethasone, on adult stem cell differentiation [36]. In order to distinguish the dual effect due to the chemical composition of the osteogenic medium and that elicited by high frequency mechanical vibration, BMSCs were cultured in osteogenic medium. In order to understand the level of differentiation toward bone tissue, the following analyses were performed: Alizarin Red test to evaluate the level of calcium deposition, molecular biology tests to estimate the expression of some of the more important osteogenic genes, and protein content analysis to quantify the amount of proteins actually translated. To study the effects of HFV on BMSC proliferation, we followed cell growth for one week using the xCELLigence system, both for control and treated cells. In addition, we performed the XTT test (cell proliferation) and direct cell counts after treatment; finally, we evaluated apoptosis in these cells with the fluorescence based-technique, ApopTag.

The final goal of the study was to develop a standard method to expedite adult BMSC differentiation towards bone tissue.

## 2. Materials and Methods

### 2.1. Isolation, Expansion, and Culture of hBMSC

Iliac crest bone marrow aspirates were collected from healthy donors after obtaining signed consent. The experimental protocol was approved by the University Ethics Committee. Mononuclear cells were isolated from bone marrow aspirates (30 mL) by density gradient centrifugation in Ficoll (density, 1.077 g/mL) (Lymphoprep, Nycomed Pharma) and plated in noncoated 75–175 cm^2^ polystyrene culture flasks (Corning Costar, Celbio, Italy) at a density of 160,000 cells/cm^2^. The culture media was supplemented with 2 mM L-glutamine, 50 *μ*g/mL gentamycin, and 5% MesenCult (Stem Cell Technologies). Cultures were maintained at 37°C in a 5% CO_2_ humidified atmosphere. After 48 h, nonadherent cells were discarded and culture medium was replaced twice a week. After reaching 80% confluence, the cells were harvested and replated for expansion at a density of 4,000 cells/cm^2^ until the 5th passage. The colony-forming unit-fibroblast assay (CFU-F) was performed as described [37]. CFU-F formation was examined after 12 days of incubation in a humidified atmosphere (37°C, 5% CO_2_); the clonogenic efficiency was calculated as the number of colonies per 10^6^ bone marrow mononuclear cells seeded. According to the International Society for Cellular Therapy on the nomenclature of mesenchymal progenitors, the cells cultured for this study were defined as multipotent stromal cells. To phenotypically characterize hBMSC and to define their purity, FACS analysis was performed on cells at different passages [37] (P3, P7, P13, and P22). After reaching 80% confluence, the cells were harvested and replated for expansion at a density of 2.5 × 10^4^ cells/cm^2^. The cells were cultured at 37°C with 5% CO_2_, three-fifths of the medium was renewed every three days, and then the cells were routinely trypsinized, counted, and used for the following experiments. We harvested the cells after four passages.

#### 2.1.1. Cell Immunophenotyping

Around 1.5 × 10^5^ cells were trypsinized to obtain a cell suspension, then incubated 20 minutes in the dark at room temperature with 10 *μ*l of fluorochrome-conjugated (FITC or PE) monoclonal antibody (BD Pharmingen and MACS). Cells have been analyzed for haematopoietic (CD34, CD45) and mesenchymal (CD90, CD105, CD13, and CD44) markers at different passages (P3, P7, P13, and P22) to define the purity at the beginning of the culture and during the culture period. After incubation with the specific antibody, cells were washed and analysed with FACS-caliber instrument (Becton Dickinson; BD, Heidelberg, Germany).

#### 2.1.2. Culture Media

We used two culture media: proliferation medium to expand adult stem cells and osteogenic medium to promote differentiation of BMSC to bone tissue.

DMEM was utilized for the proliferation medium (Dulbecco's Modified Eagle's Medium) (EuroClone, Milan, Italy), to which we added 1% Hepes, 2% NaPyruvate, 1% Antibiotics (Penicillin/Streptomycin), and 5% Mesenchymal Stem Cell Stimulatory Supplement (StemCell Technologies, Vancouver, BC, Canada). This culture medium was used to expand BMSC.

For the osteogenic medium, DMEM (Dulbecco's Modified Eagle's Medium) (EuroClone, Milan, Italy) was utilized, supplemented with 1% Hepes, 2% NaPyruvate, 1% Antibiotics (Penicillin/Streptomycin), 15% Osteogenic Stimulatory supplement (StemCell Technologies, Vancouver, Canada), 10^−8^ M Dexamethasone, 50 *µ*g/mL Ascorbic Acid, and 3.5 mM *β*-Glycerophosphate (all reagents were obtained from StemCell Technologies, Vancouver, Canada).

### 2.2. The Bioreactor

**Figure 1:**
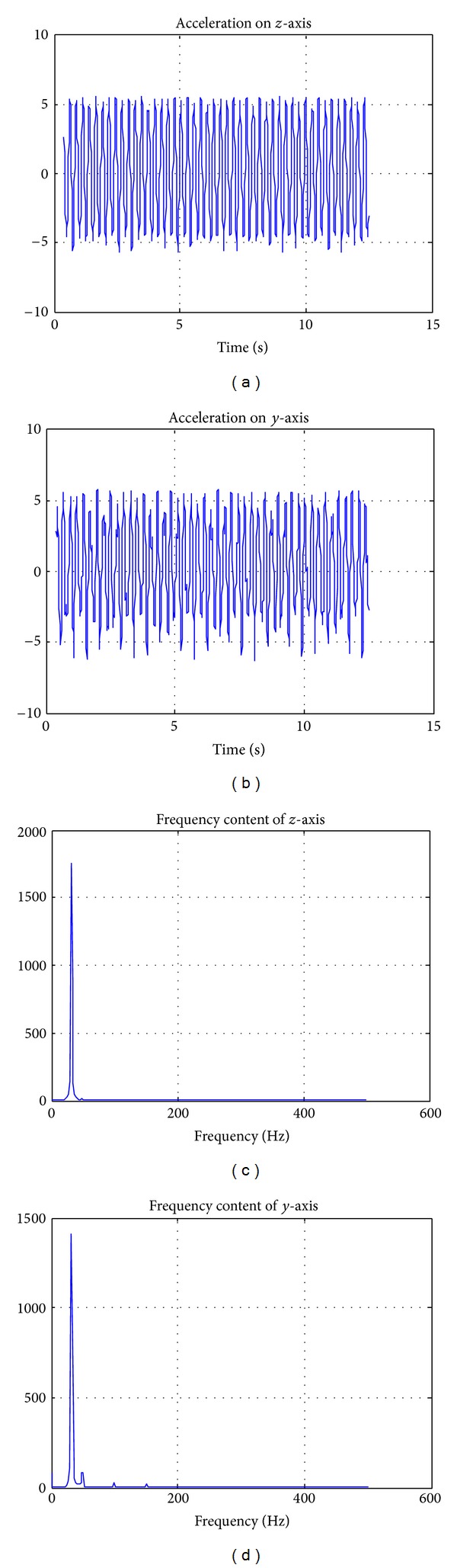
Detection of acceleration signals plotted with Matlab 7.1. The signals are shown on the *z*-axis (a), *y*-axis (b) and in the frequency domain after a Fourier Transform for *z*-axis (c) and *y*-axis (d). The measure unit of the acceleration is m/s^2^. No significant acceleration is recorded on the *x*-axis, that is, along the rotational axes of the motor (see Figure 2).

**Figure 2:**
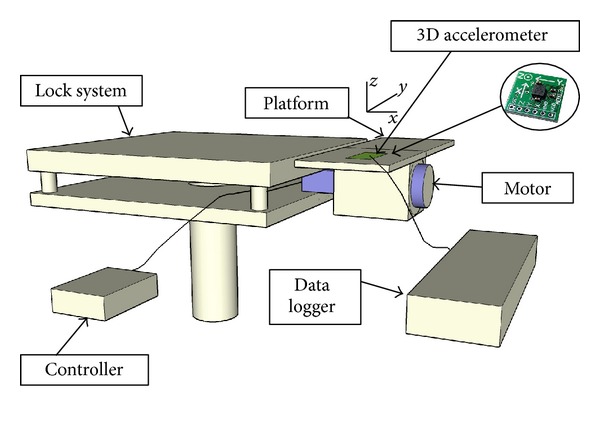
Representation of the bioreactor and its axes using SketchUp 5. The system is constituted by a locking block that anchors the motor and its attached platform to the earth reference system. The cell culture wells are plated on the top of the platform and fixed on it, together with the triaxial accelerometer to record the acceleration perceived by the cells. A controller directs the motor performance.

To stimulate the cells, a previously described [38] custom made “bioreactor” was used. The device is composed of a platform coupled to an eccentric, voltage-controlled DC motor, which works between 1 and 120 Hz with a voltage range of 2.5–24 V. Frequency, mode, and duration of the stimulus can be set with an electronic controller. The dishes and flasks were secured to the platform together with a triaxial accelerometer anchored to the upper layer of the platform as described in Figure 2, which records the acceleration utilized to stimulate the cells on the 3 axes. Accelerometer results are presented in Figure 1, where it is clear that the movement created by the motor on the platform (and the cell plate itself, since it constitutes a single solid system with the platform) operates only on two axes. The bioreactor stimulates the cells by applying a vibration in the vertical plane, perpendicular to the rotation axis, with a peak acceleration of around 5.8 m/s^2^ on both the *y* and *z* axes (Figure 2 illustrates the bioreactor and its axes).

### 2.3. Bioreactor Cultures

In order to evaluate the effects of vibration on BMSC differentiation toward bone tissue, we performed the experiments in triplicate. We stimulated the cells for 21 days and 40 days, both for 45 minutes a day at 30 Hz. These parameters were chosen based on the results of previous experiments on the effects on bones after 30 Hz of whole body stimulation [39, 40] and our results on the osteoinductive effects of the same stimulation pattern on SAOS-2 [19] and hADSCs [20]. Once BMSCs reached 80% confluence, they were trypsinized and plated at a density of 5.000 cells/cm^2^ in 9 cm dishes and then divided into two groups: one subjected to mechanical treatment in osteogenic medium (T) and one in osteogenic medium, without any mechanical treatment (C).

For each experiment, the medium was refreshed every three days. We processed three samples for each group, and the experiment was repeated three times to increase statistical confidence.

### 2.4. Alizarin Red Test

The Alizarin Red test was used to determine the presence of calcium deposition, an indicator of the osteogenic lineage [41, 42]. This is an early-stage marker of matrix mineralization, which indicates a crucial step towards the formation of calcified extracellular matrix associated with true bone. The cells were stained with pH-adjusted (4.1–4.3) 2% Alizarin Red solution (Electron Microscopy Sciences, Fort Washington, PA, USA), washed, and then photographed using transmitted light.

The stain was eluted by adding 1 mL 10% cetylpyridinium chloride per well for 10 min at room temperature with gentle agitation. Afterwards, the intensity of the color, proportional to the calcium deposition, was measured with the Nanodrop system (Nanodrop Technologies, Wilmington, USA) at a wavelength of 562 nm.

### 2.5. Molecular Biology Assays

In order to evaluate the extent of osteogenic protein transcription, we measured the expression of some of the more important osteogenic genes which are indicators of human BMSC differentiation toward bone: OP (osteopontin), RUNX2 (Runt-related transcription factor 2), ALP (Alkaline Phosphatase), and BOSP (Bone Sialoprotein). Gene expression was normalized against the housekeeping gene GAPDH (glyceraldehyde-3-phosphate-dehydrogenase), using quantitative real-time PCR. The genes analyzed in our study are extensively characterized genes used to evaluate stem cell bone differentiation. In particular, RUNX2 is required for the determination of the osteoblast lineage [43–46]. The protein level of RUNX2 in osteoblasts reduces during bone development, and osteoblasts acquire mature phenotypes, which are required for mature bone formation. Regarding OP and BOSP, these represent the late phase of differentiation towards the osteoblastic phenotype and they are widely used in gene expression analyses [45, 47]. ALP is also an important phenotypic marker of osteoblast differentiation and is expressed in the early stage of bone formation [48].

#### 2.5.1. RNA Extraction and Synthesis of c-DNA

In order to evaluate gene expression, RNA was extracted from treated and untreated cells with the Qiagen RNeasy Micro kit, immediately at the end of each experiment. Cells were suspended in *β*-mercaptoetanol and lysis buffer, frozen in liquid nitrogen, and stored at −80°C until extraction was performed. RNA extraction included the addition of 70% ethanol and DNase to increase purity. Afterwards, total extracted RNA was retrotranscribed into c-DNA, with the iScript cDNA Synthesis kit (Bio-Rad, Hercules, CA, USA).

#### 2.5.2. Quantitative Real-Time RT-PCR

To check the quantitative expression of the investigated osteogenic genes, Quantitative real-time RT-PCR was performed with the Mini-Opticon Real-Time PCR System (Bio-Rad, Hercules, CA, USA). Gene expression was analyzed in triplicate and normalized to GAPDH gene expression. Analysis was performed in a total volume of 20 *μ*L amplification mixture containing 2 x (10 *μ*L) Brilliant SYBR Green QPCR Master Mix (Stratagene, USA), 2 *μ*L cDNA, 0.4 *μ*L of each primer, and 7.2 *μ*L H_2_O. Thermal cycling was initiated by denaturation at 95° for 3′, followed by 40 cycles at 95° for 5′′ and 60° for 20′′.

To perform the real-time analysis, we used Invitrogen (Carlsbad, CA, USA) primers for the genes listed in Table 1.

### 2.6. Proliferation Tests

In order to analyze the effect of HFV on BMSC proliferation, we performed four tests: xCELLigence analysis, the XTT test (proliferation), direct cell count (Burker chamber), and the apoptosis analysis with the ApopTag kit.

#### 2.6.1. xCELLigence System

A real-time cell analyzer (RTCA) SP (Roche) was used to supervise proliferation level of BMSC treated or untreated with HFV. (Xing et al. [49], Roche Diagnostic GmbH, 2008.) The RT-CES system is a real-time cell analyzer developed by Roche; it includes an electronic sensor analyzer, a device station, and a 96-well e-plate. The e-plate is a standard flat-bottom 96-well culture plate, with circle-on-line sensor electrode arrays incorporated in each well. The device station, containing the e-plates, is placed in the incubator and connected to the electronic sensor analyzer via electrical cables. The electronic impedance of sensor electrodes is measured to allow monitoring of cell changes; this measure provides the cell index parameter. The cell index (CI) is calculated by dedicated software as the difference between the measure of impedance at T0 (media alone) and one measurement at each time point. This CI is a dimensionless value that is correlated to cell number and/or viability. Under the control of the RT-CES software, experimental data are measured automatically by the sensor analyzer. The analysis of BMSC proliferation was performed in 12 replicates. We seeded 5.000 cells/cm^2^ in 180 *μ*l DMEM 10% FCS in 96-well e-plates. For each pair of 96-well e-plates, one was treated for one week at 30 Hz 45′/day, and one was left untreated as a control. Cell proliferation was monitored for one week, with an impedance measurement every minute. The starting density used was the optimal density for human cell line growth.

#### 2.6.2. Apoptosis Analysis

Apoptosis was tested with ApopTag Fluorescein In Situ (Chemicon) for both treated and untreated BMSC. This technique reveals apoptosis by specifically detecting DNA cleavage and chromatin condensation. The ApopTag kit modifies fragmented genomic DNA utilizing terminal deoxynucleotidyl transferase (TdT) for the detection of positive cells by specific staining. TdT catalyzes the template-independent addition of nucleotide triphosphates labelled with Digoxigenin to the 3′-OH ends of double-stranded or single-stranded DNA. Finally, using FITC-labelled antidigoxigenin antibody, apoptotic cells are stained and visualized with a fluorescent microscope.

#### 2.6.3. XTT Test

The XTT assay was used as a standard method to evaluate cell proliferation [50, 51]. This spectrophotometry-based test reveals cellular metabolism, a strong indicator of an increase in cell number.

To realize this test, 5.000 cells/cm^2^ were plated on a 24-well plate, with an area of 1.76 cm^2^/well. After the last treatment at day 7, the XTT reagent (Roche kit, Basel, Switzerland) was added to each well (treated and untreated). The reagent was left to react for four hours and afterwards an ELISA system was used to read light absorbance at 562 nm. Light absorbance is proportional to metabolic activity of the cells; the resulting data reflect the effect of the vibrating treatment on treated and untreated BMSCs proliferation.

#### 2.6.4. Cell Counting

The ratio of proliferation for treated and untreated BMSCs was also evaluated by directly counting cells with a Burker chamber. The cells were plated in 12 wells of a 24 well-plate with an area of 1.76 cm², 5.000 cells/cm^2^, stimulated at 30 Hz and stopped at day 7 of treatment. Cells were detached with Trypsin, resuspended in an appropriate volume of PBS, and counted in a Burker chamber.

### 2.7. Protein Content Analysis

To evaluate the amount of proteins actually translated, we measured the levels of the more important osteogenic proteins (collagen I, collagen III, osteocalcin, human decorin, osteopontin, alkaline phosphatase, osteonectin, and bone sialoprotein), commonly used to test the level of bone differentiation [52].

#### 2.7.1. DNA Content

On days 21 and 40, culture dishes were extensively washed with PBS. The cells were then lysed by a freeze-thaw method in sterile deionised distilled water. The released DNA content was evaluated with a fluorometric DNA quantification kit (PicoGreen; Molecular Probes, Eugene, OR, USA). DNA obtained from a known number of undifferentiated or differentiated BMSCs was used to create standard curves, [52]. From these curves, we could extrapolate the number of cells per dish.

#### 2.7.2. Rabbit Polyclonal Antisera

The rabbit polyclonal anti type I and III collagen, antidecorin, antiosteopontin, antiosteocalcin, antiosteonectin, and antialkaline phosphatase were provided by Dr. Larry W. Fisher (National Institutes of Health, Bethesda, MD, USA).

For polyclonal antibody production against human fibronectin (HFn), New Zealand rabbits were injected intraperitoneally five times at 12-day-intervals with 100 *μ*g of the purified HFn [52]. The antigen was emulsified with an equal volume of complete Freund's adjuvant for the first immunization followed by four injections with incomplete adjuvant. The rabbit was bled, and the serum was tested for reactivity to the purified HFn using an ELISA assay. The specific anti-Fn IgGs were purified by affinity chromatography on protein G-Sepharose columns according to the manufacturer's recommendations (Amersham Biosciences, Piscataway, NJ, USA). Antibody titers were assayed by ELISA.

#### 2.7.3. Set of Purified Proteins

Decorin, type-I collagen, and fibronectin were purified as described previously [52]. Osteocalcin was acquired from Biomedical Technologies, Inc. (Stoughton, MA, USA); osteopontin and osteonectin were obtained from Assay Designs, Inc. (Ann Arbor, MI, USA); type-III collagen and alkaline phosphatase were purchased from Sigma-Aldrich, Inc.

#### 2.7.4. Extraction of the Extracellular Matrix Proteins from Cell Cultures and ELISA Assay

On days 21 and 40, in order to evaluate the amount of extracellular matrix constituents, the dishes were washed extensively with sterile PBS to remove the culture medium and then incubated for 24 h at 37°C with 4 mL sterile sample buffer (20 mM Tris-HCl, 4 M GuHCl, 10 mM EDTA, 0.066% (w/v) SDS, and pH 8.0). Sample buffer aliquots were removed and the total protein concentration in the culture systems was evaluated with the BCA Protein Assay Kit (Pierce Biotechnology, Inc., Rockford, IL, USA).

Calibration curves to measure type-I and -III collagens, decorin, osteopontin, osteocalcin, osteonectin, fibronectin, and alkaline phosphatase were performed. Microtiter wells were coated with increasing concentrations of each purified protein, from 10 ng to 2 *μ*g, in coating buffer (50 mM Na_2_CO_3_, and pH=9.5) overnight at 4°C. Control wells were coated with bovine serum albumin (BSA) as a negative control. In order to measure the extracellular matrix amount of each protein by the ELISA assay, microtiter wells were coated overnight at 4°C, with 100 *μ*L of the previously extracted extracellular matrix (20 *μ*g/mL in coating buffer). After three washes with PBST (PBS containing 0.1% (v/v) Tween 20), the wells were blocked by incubating with 200 *μ*L PBS containing 2% (w/v) BSA for 2 h at 22°C. The wells were subsequently incubated for 1.5 h at 22°C with 100 *μ*L of the antitype I and III collagens, antidecorin, antiosteopontin, antiosteocalcin, antiosteonectin, antialkaline phosphatase rabbit polyclonal antisera (1 : 500 dilution in 1% BSA). The same dilution was used for the anti-Fn rabbit polyclonal IgG. After washing, the wells were incubated for 1 h at 22°C with 100 *μ*L of HRP-conjugated goat anti-rabbit IgG (1 : 1000 dilution in 1% BSA). The wells were finally incubated with 100 *μ*L of development solution (phosphate-citrate buffer with *o*-phenylenediamine dihydrochloride substrate). The color reaction was stopped with 100 *μ*L of 0.5 M H_2_SO_4_, and the absorbance values were measured at 490 nm with a microplate reader (BioRad Laboratories). An underestimation of absolute protein deposition is possible because the sample buffer, used for matrix extraction, contained sodium dodecyl sulphate, which may interfere with protein adsorption during the ELISA assay. The amount of extracellular matrix constituents throughout all the samples is expressed as fg/(cell × dish) and as the ratio between treated and control samples.

### 2.8. Statistical Analysis

Results are expressed as the mean and (standard deviation) SD. To evaluate the effects on the treated groups with respect to controls, a two-tailed Student's *t*-test was performed. In particular, for the Alizarin results and gene expression results unpaired *t*-tests were used, while for the xCELLigence test a paired *t*-test was performed. Statistical significance was established at *P* ≤ 0.05, for which *P* is the null hypothesis.

## 3. Results

### 3.1. BMSCs Show Mesenchymal Markers at Different Passages

FACS analysis revealed that BMSCs at different passages (P3, P7, P13, and P22) possess the same surface markers expression of mesenchymal stem cells. Cells show simultaneous expression of cell surface mesenchymal markers (>97% of cells were positive) as CD13, CD90, CD105, and CD44 with a concomitant absence of haematopoietic markers CD45 and CD34 (<6% of cells were positive) (data not shown). This expression pattern represents a specific phenotype for cultured MSC. So, BMSCs stimulated with HFV are effective mesenchymal stem cells.

### 3.2. At 40 Days HFV Increases the Level of Calcium Deposition in Treated BMSC

**Figure 3:**
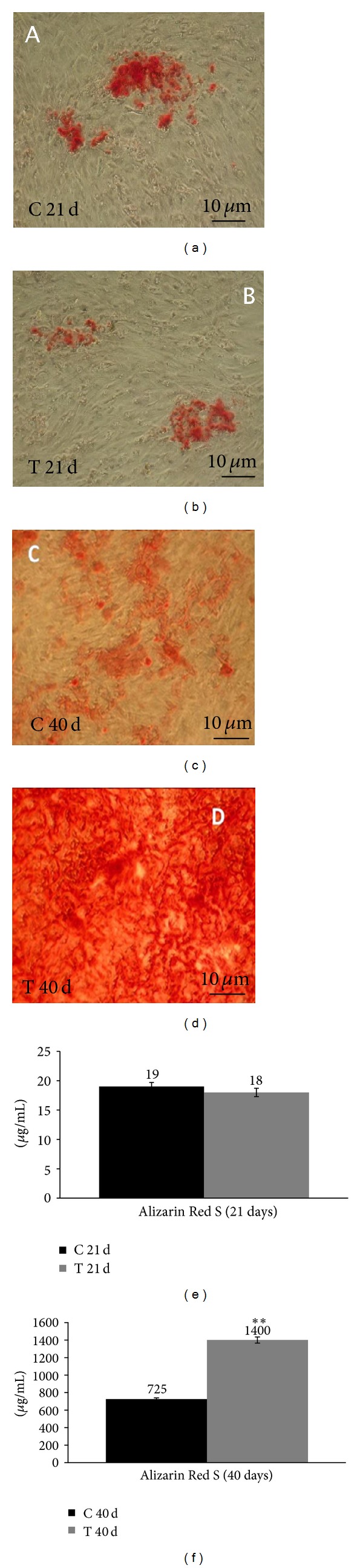
Alizarin Red S results in BMSCs. (a) Control sample at 21 days, 10 x magnification. (b) Treated sample at 21 days, 10 x magnification. (c) Control sample at 40 days, 10 x magnification. (d) Treated sample at 40 days, 10 x magnification. (e) Calcium deposition plots at 21 days. (f) Calcium deposition plots at 40 days. The scale bars are equivalent to 10 *μ*m. The data are presented as the mean and standard deviation (SD). Statistical significance values are indicated as *0.05 > *P* > 0.001 and **P < 0.001. The unpaired *t*-test was used to evaluate data significance.

The results of the Alizarin Red test for 21- and 40- day cultures of treated and untreated BMSC are provided in Figure 3. BMSC treated for 21 days with HFV in osteogenic medium (Figure 3(e)) presented a slightly higher concentration of calcium deposition in the control sample with respect to the treated one (18.5 and 19.5 *µ*g/mL, resp.), although the difference was not statistically significant. The effect was more evident at 40 days (Figure 3(f)): the alizarin red concentration in the treated sample was 1400 *µ*g/mL, while in the control sample it was 725 *µ*g/mL (*P* < 0.001). The 10 x magnification images (Figures 3(a), 3(b), 3(c), and 3(d)) represent an example of the fields used to evaluate the histological presence of calcium deposition in BMSC. The positive effects of the combination of osteogenic medium and mechanical treatment on treated BMSC (Figures 3(c) and 3(d)) are evident and they indicate the differential effect of the treatment.

### 3.3. Gene Expression Results in Osteogenic Medium: HFV Enhances RUNX2, OP, and BOSP Gene Expressions in BMSC

**Figure 4:**
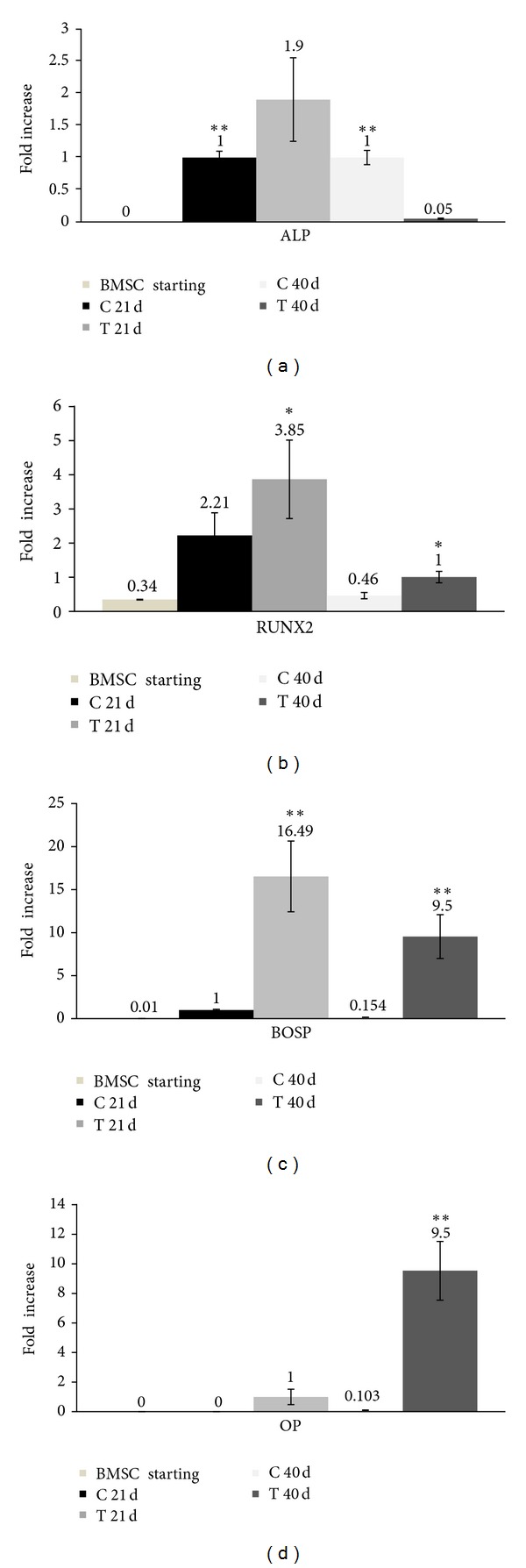
BMSC gene expression at the beginning of the stimulation (BMSC starting) and at 21 and 40 days. (a) qRT-PCR for ALP. (b) qRT-PCR for RUNX2. (c) qRT-PCR for BOSP. (d) qRT-PCR for OP. Results are normalized with reference to the housekeeping gene (GAPDH). Statistically significant values are indicated as *0.05 > *P* > 0.001 and **P < 0.001. The unpaired *t*-test was used to evaluate data significance.

The results on the expression of ALP, RUNX2, BOSP, and OP in osteogenic medium after 21 and 40 days culture in control and treated BMSCs are presented in Figure 4. It was also investigated the expression of the same genes on BMSC prior to cells being differentiated and treated with HFV, and we observed the expression of RUNX2 gene only (Figure 4(b)), that is, the master gene of osteogenic differentiation.

The expression of the gene that encodes for ALP at 21 days was 0.9-fold higher in the treated sample with respect to the control (Figure 4(a), *P* = 0.005); while at 40 days, ALP expression in the control sample was double with respect to the treated sample (*P* = 0.005). HFV enhanced BOSP, OP, and RUNX2 expression in all treated samples with respect to controls (Figures 4(b), 4(c), and 4(d)). In particular, at 21 days, RUNX2 expression was 1.64-fold higher in treated cells with respect to controls (Figure 4(b), *P* = 0.005), BOSP expression was 16.49-fold higher (Figure 4(c), *P* < 0.001), and OP was detected only in the treated sample and not in the control (thus, the difference between the two samples and the *P* value could not be calculated). It can be hypothesized that HFV served as a stimulus in osteogenic medium at 40 days. In this medium after 40 days, the process of calcium mineralization was in an advanced phase, as confirmed by the Alizarin Red test results (Figure 3). In fact, the expression of RUNX2 in the treated sample was 0.54-fold higher than the control (Figure 4(b)), with *P* = 0.003, and the two genes characterizing a later differentiation phase were expressed at higher levels: both osteopontin and bone sialoprotein were 9.5-fold higher (Figure 4(d), *P* < 0.001) in treated samples with respect to controls.

### 3.4. Protein Content Analysis in Treated and Untreated BMSCs

The total protein concentration was measured after 21 and 40 days culture: at 21 days the total protein concentration was 0.847 mg/mL for the untreated sample and 0.908 mg/mL for cells treated with HFV. At the end of the culture period (40 days), protein content was 0.861 mg/mL and 0.942 mg/mL, respectively, for the control and stimulated samples.

In order to evaluate the amount of extracellular matrix constituents produced by the cells, an ELISA assay was performed. In Table 2, the protein content results are presented for treated and control samples in osteogenic medium at 21 and 40 days, as fg/(cells × dish). At 21 days and at the end of the culture period (40 days), the deposition of bone proteins in HFV stimulated samples was considerably enhanced (*P* < 0.05) in comparison with the control samples. The enhancement of protein deposition was evident especially for alkaline phosphatase (ALP), osteocalcin (OC), and osteopontin (OP): for ALP it was about 1.2-fold and 1.6-fold for 21 and 40 days, respectively, for OC 1.9-fold (21 days) and 1.86-fold (40 days), and for OP 1.7-fold (21 days) and 1.6-fold (40 days) (Table 2).

### 3.5. HFV Decreases the Proliferation Rate in BMSC

**Figure 5:**
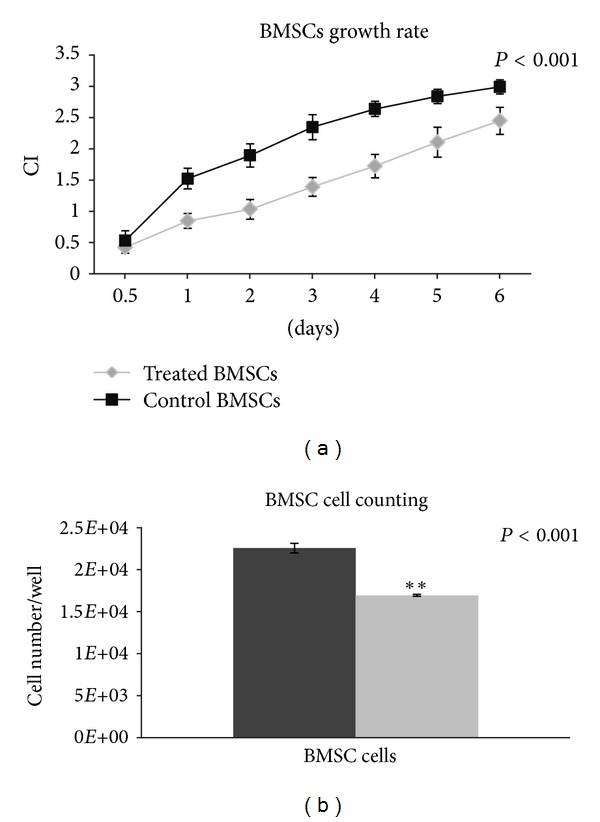
xCELLigence and cell counts. (a) Growth comparison graph for treated and untreated BMSCs after one week of culture. (b) Representation of the number of control and treated BMSC. Statistically significant values are indicated as *0.05 > *P* > 0.001 and **P < 0.001. The paired *t*-test was used to evaluate significance of the xCELLigence data.

Data extrapolated with the xCELLigence system are shown in Figure 5(a). Black lines represent control cells and gray lines treated cells. Treated BMSCs displayed a lower rate of proliferation with respect to control cells for all time points. The cell index, which is a quantitative measure of the cell number present in a single well, was always significantly lower (*P* < 0.001, Figure 4(a)) in treated cell wells with respect to controls. At the end of the 6th day, treated and control cells tended to plateau. Nevertheless, XTT results revealed no statistically significant differences between control and treated BMSCs (data not shown), even if BMSC numbers performed at the end of the vibration period were significantly lower in the treated samples with respect to the control (Figure 4(b), *P* < 0.001), indicating a lower proliferation rate for cells subjected to HFV. We could assume that treated cells slowdown their cell cycle, as revealed by xCELLigence and cell counts, but maintain their vitality as metabolism was not altered by the stimulation.

**Figure 6:**
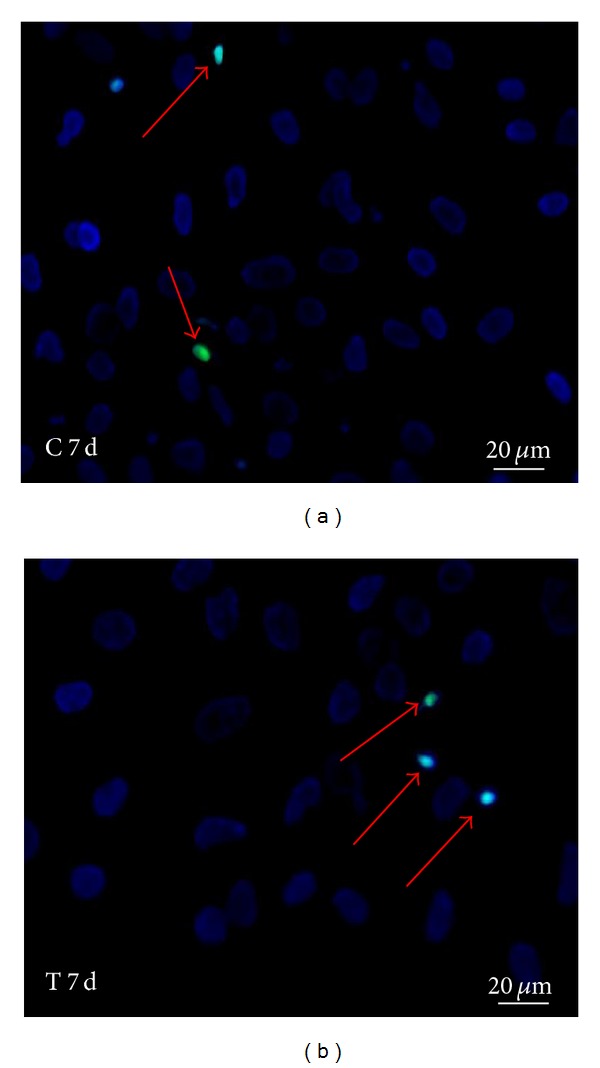
ApopTag Fluorescein Direct In Situ technique on cells at 7 days. (a) Picture of ApopTag technique on C BMSC at day 7. The arrows indicate pyknotic nuclei of cells, with respect to other nuclei counterstained with DAPI. (b) Picture of ApopTag technique on T BMSC with HFV at day 7. The arrows indicate pyknotic nuclei of cells.

Since a reduction in the proliferation rate could be due to apoptosis induced by the treatment, we analysed both treated and untreated BMSCs with the ApopTag Fluorescein In Situ technique, a method that detects apoptotic cells *in situ* by the indirect TUNEL method. Analyses of cells treated for 7 days did not reveal statistical differences in the percentage of apoptotic cells with respect to untreated cells. In fact, treated BMSCs had an average of 3.2% (*P* = 0.001) apoptotic cells with respect to 2.6% (*P* = 0.001) apoptotic cells in control samples (Figures 6(a) and 6(b)). From these results, we can infer that HFV slows down BMSC proliferation without, however, inducing apoptosis. It is possible that the effect of mechanotransduction induced by the HFV produces alterations in the cytoskeleton that slowsdown cycling of treated cells. Further analyses are necessary to clarify this aspect of treatment.

## 4. Discussion

Vibration is a mechanical treatment widely utilized in clinical and rehabilitation centres. In fact, in recent years application of high-frequency vibration in clinical trials has greatly increased for osteoporosis, stroke recovery, Parkinson's disease, arthritis, fibromyalgia, cystic fibrosis, and obesity [53–56]. In addition, many studies have elucidated the effects of vibration at the tissue and cellular levels. In particular, we demonstrated the inductive effect of high-frequency vibrations on the differentiation toward bone for the SAOS-2 cell line and hASC [19, 20]. The aim of this study was, therefore, to test *in vitro* the enhancing effect of the same stimulation on BMSC proliferation and differentiation in the presence of an osteogenic medium. BMSCs have already demonstrated the capacity to spontaneously differentiate into bone on scaffolds [57] or with chemical [58, 59] or mechanical [60] stimulation. In preliminary studies, we have also tested the effects of HFV on BMSC in proliferative medium (without osteoinductive agents), such as in hASC experiments [20], to analyze the osteoinductive effect of the mechanical stimulation itself on cells. In fact, *in vitro* studies showed that HFV has important effects on differentiation and proliferation [61, 62]. Cells are able to respond to the extracellular matrix by interpreting the physical, chemical, and topographical properties of the substrate [63]. This interaction of cells with their substratum is called ‘‘mechanosensing” [64]. External forces, shear stress, or mechanical loading (such as HFV) may modify the cytoskeleton elements of cells, activating “mechanoreceptors.” These mechanosensors in turn activate a cascade of secondary messengers that determine structural rearrangements deep within the cytoplasm and nucleus of cells [64, 65].

It is clear that bone cells and stem cells respond to the mechanical effects of high-frequency vibration, but in this study we did not attempt to increase the effects of shear stress or vibration stress on cell deformations, but rather we focused on the osteoinductive and proliferative effects of vibration in adult mesenchymal stem cells.

We found that HFV itself was not sufficient to promote any statistical differences between control and treated cells, either in gene expression or ECM protein content. Therefore, since the inductive properties of osteogenic medium have already been demonstrated, we separately studied the effects of high-frequency vibration treatment on BMSC in osteogenic medium. In this manner, we tested the effects of high-frequency vibration in enhancing differentiation towards bone tissue in comparison with control cells in osteoinductive medium only.

The results obtained demonstrate the strong differentiating effect of HFV treatment. In fact, previous studies on BMSC differentiation towards bone tissue showed that osteogenic medium had a significant effect on the commitment of cells to pursue the osteoblastic phenotype. The culture of stem cell clones in osteogenic medium is essential to mimic the microenvironment that the cell experiences during *in vivo* bone formation [66]. The action of osteoinductive agents to promote stem cell differentiation has been known since 1988, when Owen and Friedenstein showed that specific factors added to culture medium may activate osteogenesis in a range of multipotent and more committed precursors [67].

The results on the deposition of calcium at 21 days indicate that high-frequency vibration treatment does not have any effect on this parameter (Figures 3(a), 3(b), and 3(e)). However, cells cultured at 21 days are in early differentiation; thus it was probably too early to highlight significant differences in terms of histology and morphology of calcium deposition.

To understand the effects of mechanical treatment on calcium deposition, it is important to evaluate the difference between treated and control samples in the mineralization phase. After 40 days, the results showed the positive effects of the combination of osteogenic medium and mechanical treatment on BMSC (Figures 3(c), 3(d), and 3(f)), the first strong indication of the differentiative effect of HFV treatment. To confirm these results, it was essential to analyze the consequences of treatment on the expression of osteogenic genes and subsequent protein translation.

The results of the (q) Real-time PCR for genes characterizing the late phase of differentiation (40 days) showed that mechanically treated samples in osteogenic medium expressed higher values of bone sialoprotein and osteopontin compared to controls in the same medium (9.5- and 9.5-fold, resp.). Also RUNX2, which is an important transcription factor associated with osteoblast differentiation [44–46], was 0.54-fold higher in treated cells with respect to controls (Figure 4(b)), but ALP, which is an early-stage marker, decreased in treated samples (Figure 4(a)). Moreover, the effect at 21 days was evident: RUNX2 was 1.64-fold higher in the treated samples (Figure 4(b)) and BOSP was 16.49-fold higher (Figure 4(c)). OP at 21 days in osteogenic medium was expressed only in the treated sample (Figure 4(d)). Bone sialoprotein and osteopontin are, in fact, expressed in the late phase of differentiation. On the contrary, alkaline phosphatase and RUNX2 are early-stage markers.

The only gene that appeared to decrease as a result of the mechanical treatment was ALP. The data at 40 days indicated that the expression of the gene encoding for ALP was strongly reduced in the treated sample (Figure 4(a)). The gene that encodes for ALP, which is expressed in the early stage of differentiation, was weakly expressed at 21 days in the treated sample, but afterwards (at 40 days) it was likely already translated into protein.

Thus, a plausible explanation is that vibration treatment stimulates early osteogenesis and, therefore, ALP has already been translated into protein and its expression is inhibited in cells that are in an advanced differentiation state. To better understand results obtained with the Alizarin Red test and molecular biology tests, we measured, with an ELISA assay, the concentration of extracellular matrix proteins in treated and untreated BMSCs (Table 2). 

The deposition of type I collagen was quite similar at 21 and 40 days of culture, where an approximate 1.4 (21 days)- and 1.3 (40 days)-fold increase was observed for HFV-exposed cells. Bone type I collagen synthesis is known to be upregulated at the proliferation stage when osteoblasts are not confluent and downregulated at subsequent stages [44]. A slight increment in both type III collagen and decorin quantity was also detected in treated samples; both of these proteins are known to be associated with type I collagen [46]. The protein increment was higher for decorin at a longer incubation time in treated samples. Decorin is important because it plays a major role in the lateral growth of collagen fibrils. Osteopontin, osteocalcin, and osteonectin, and other recognized fundamental bone matrix constituents, were shown to be deposited by treated BMSC at a higher level after 21 days of incubation (*P* < 0.05) (Table 2). Deposition of the previous indicated proteins was significantly higher at longer incubation times (*P* < 0.05) (Table 2). All of these extracellular matrix proteins are organic components of bone and are implicated in bone formation and remodelling. Osteopontin is known to play an important role in cell attachment and calcification of mineralized tissue [45], whereas osteocalcin is the most recent secreted extracellular matrix protein identified [68]. Osteonectin is a calcium- and collagen-binding ECM glycoprotein and also acts as a modulator of cell-matrix interactions [69]. The quantity of fibronectin is not completely reliable because contamination due to the presence of serum in the culture medium is possible; however, these data were quite significant. Fibronectin has been reported to promote both cell adhesion and proliferation in many cell types [70]. In view of this, the important increases in *in vitro* protein levels of ALP (makes phosphate available for calcification), osteopontin (anchors bone cells via their *α*V*β*3 integrin to the mineralized bone surface), osteonectin (functions as a modulator of cell-matrix interactions), osteocalcin (a protein marker of bone differentiation), and type I collagen (the major organic component of bone matrix produced by osteoblasts) are quite important. All together, these results suggest that the main effect of HFV was enhanced osteoblast differentiation and the promotion of bone ECM deposition. These data are “in sync” with the Alizarin Red test results showing the increase in calcium deposition at 40 days.

Finally, proliferation tests in treated and untreated BMSCs indicated that HFV slows down cell proliferation (Figures 4(a) and 4(b), *P* < 0.001), but without activation of apoptotic processes. The XTT test did not reveal any differences between control and treated cells (they had the same viability), and the apoptosis process was not induced; these cells were metabolically active. HFV most likely interferes with the cell cytoskeleton, disturbing the cell cycle of treated cells. It cannot be excluded, however, that these processes are induced by different mechanotransduction mediators, such as the shear stress caused by the movement of the medium induced by the vibration. Further analyses will be necessary to study the possible synergistic effects on the cells.

Although these encouraging findings indicate that high-frequency vibration treatment with an acceleration peak range around half g accelerates differentiation of BMSC toward bone, other tests should be carried out.

The applications of mechanical vibration are widespread in the clinical setting, particularly in sports training and medicine. Vibrations, transmitted along the body during locomotion, are responsible for maintaining posture, muscle tone, and bone remodeling, and recently their effectiveness in some murine models of bone disease has been demonstrated [71, 72]. Nevertheless, *in vivo* results remain controversial, as important variables, like nutrition and genetic make-up, may impact response to HFV stimulation. This study should be considered a preliminary *in vitro* study to set up further analyses on the effect of HFV treatment on cells and subsequently to be translated to animal and human trials. The possible clinical application of this investigation is related to a faster induction of the differentiation of patient-derived BMSCs into osteoblasts and osteocytes, that, in association with a proper scaffold, could generate an autologous bone graft ready to be implanted in the patient in a reasonable time frame.

## Figures and Tables

**Table 1:** List of the primers used for the (q) real-time PCR.

Genes	FW	RW
ALP	5′ CTA TCC TGG CTC CGT GTC C 3′	5′ AGC CCA GAG ATG CAA TCG 3′
BOSP	5′ GGG CAG TAG TGA CTC ATC CG 3′	5′ TCA GCC TCA GAG TCT TCA TCT TC 3′
RUNX2	5′ ACA GTA GAT GGA CCT CGG GA 3′	5′ ATA CTG GGA TGA GGA ATG CG 3′
OP	5′ GTG ATT TGC TTT TGC CTC CT 3′	5′ GCC ACA GCA TCT GGG TAT TT 3′

**Table 2:** Normalized amount of the extracellular matrix constituents secreted and deposited by BMSC exposed or not to HFV after 21 and 40 days of culture. In the comparison with control samples, a *P* value < 0.05 was considered statistically significant (*).

	Matrix protein deposition after 21 days cell culture in fg/(cell × dish)			Matrix protein deposition after 40 days cell culture in fg/(cell × dish)		
	Control	HFV treated	HFV treated/control	Control	HFV treated	HFV treated/control
Alkaline phosphatase	11.85 ± 0.06	14.70 ± 1.21	1.2	10.10 ± 0.06	15.78 ± 1.02	1.6*
Decorin	2.61 ± 0.01	3.06 ± 0.09	1.2	2.10 ± 0.01	3.72 ± 0.11	1.7*
Fibronectin	9.72 ± 0.05	11.60 ± 0.87	1.2	4.05 ± 0.07	4.82 ± 0.97	1.2
Osteocalcin	1.39 ± 0.09	2.65 ± 0.07	1.9*	2.42 ± 0.02	4.50 ± 0.02	1.86*
Osteonectin	2.45 ± 0.01	5.49 ± 0.24	2.2*	3.08 ± 0.01	9.80 ± 3.41	3.2*
Osteopontin	3.12 ± 0.12	5.40 ± 0.32	1.7*	4.15 ± 0.12	7.10 ± 0.09	1.7*
Type I collagen	35.00 ± 1.23	49.00 ± 3.43	1.4*	36.00 ± 2.13	50.29 ± 0.93	1.4*
Type III collagen	3.50 ± 0.98	3.60 ± 0.09	1.02	4.42 ± 0.01	5.12 ± 1.03	1.15
